# A comprehensive review about the utilization of immune checkpoint inhibitors and combination therapy in hepatocellular carcinoma: an updated review

**DOI:** 10.1186/s12935-022-02682-z

**Published:** 2022-08-23

**Authors:** Faezeh Sharafi, Sadegh Abaei Hasani, Samira Alesaeidi, Mohammad Saeed Kahrizi, Ali Adili, Shadi Ghoreishizadeh, Navid Shomali, Rozita Tamjidifar, Ramin Aslaminabad, Morteza Akbari

**Affiliations:** 1 Student Research Committee, School of Medicine, Shahid Beheshti University of Medical Sciences, Tehran, Iran; 2 Cancer Research Center, Department of General Surgery, Shahid Beheshti University of Medical Science, Tehran, Iran; 3 Department of Internal Medicine and Rheumatology, Rheumatology Research Center, Tehran University of Medical Sciences, Tehran, Iran; 4 Alborz University of Medical Sciences, Karaj, Iran; 5 Senior Adult Oncology Department, Moffitt Cancer Center, University of South Florida, Tampa, Florida USA; 6 Department of Oncology, Tabriz University of Medical Sciences, Tabriz, Iran; 7 Immunology Research Center, Tabriz University of Medical Sciences, Tabriz, Iran; 8 Department of Medical Biochemistry, Faculty of Medicine, Ege University, Izmir, 35100 Turkey; 9 Department of Medical Biotechnology, Faculty of Advanced Medical Sciences, Tabriz University of Medical Sciences, Tabriz, Iran; 10 Department of Stem Cell, Institute of Health Sciences, Ege University, Izmir, 35100 Turkey

**Keywords:** Immune checkpoint inhibitors, Hepatocellular carcinoma, Combination therapy

## Abstract

A pharmacological class known as immune checkpoint inhibitors (ICIs) has been developed as a potential treatment option for various malignancies, including HCC. In HCC, ICIs have demonstrated clinically significant advantages as monotherapy or combination therapy. ICIs that target programmed cell death protein 1 (PD-1) and programmed cell death protein ligand 1 (PD-L1), as well as cytotoxic T lymphocyte antigen 4 (CTLA-4), have made significant advances in cancer treatment. In hepatocellular carcinoma (HCC), several ICIs are being tested in clinical trials, and the area is quickly developing. As immunotherapy-related adverse events (irAEs) linked with ICI therapy expands and gain worldwide access, up-to-date management guidelines become crucial to the safety profile of ICIs. This review aims to describe the evidence for ICIs in treating HCC, emphasizing the use of combination ICIs.

## Introduction

Hepatocellular carcinoma (HCC) is a malignant tumor that affects the liver and has a lousy prognosis and a high death rate [1]. Although developments have been accomplished in diagnosing and treating liver cancer in recent years, the forecast remains poor. Within 5 years after surgery, liver cancer’s recurrence and metastatic rate are as high as 70% [2]. HCC, the most frequent subtype of liver cancer, is the sixth most common malignancy and the fourth major cause of cancer-related death globally [3]. High malignancy and mortality and fast progression, recurrence, and metastasis are all characteristics of HCC. Curative hepatectomy, ablation, embolization, chemotherapy, and liver transplantation are among the therapeutic options for HCC. However, because many patients are detected late, the prognosis remains dismal in most cases [4].

In HCC, the tumor microenvironment (TME) is complex and significantly impacts tumor development and growth. Tumor cells and stromal cells engage in a variety of complex interactions, and increased levels of immunosuppressive cytokines like interleukin-10 and transforming growth factor (TGF-β) support the formation of an immunosuppressive milieu defined by immune cell dysfunction and development of immunosuppressive cells, including regulatory T (Treg) cells and cells expressing checkpoint molecules, cytotoxic T-lymphocyte antigen 4 (CTLA-4), programmed cell death 1 (PD-1), programmed death ligand-1 (PD-L1), programmed death ligand-2 (PD-L2), T cell immunoglobulin mucin domain molecule 3 (Tim-3), and lymphocyte activating gene three protein (LAG-3) [5]. In addition, long-term antigen exposure can also induce T cells to overexpress immunosuppressive checkpoint molecules, leading to cell exhaustion, immunological escape of tumor cells, and the development of HCC [6].

Immune checkpoint molecules modulate the immune response, avoiding improper immune reaction and allowing self-tolerance. Inhibitory checkpoint molecules have become critical targets for anticancer therapy because they provide a method for malignancies to avoid immune monitoring [7]. A pharmacological class known as immune checkpoint inhibitors (ICIs) has been introduced as a potential therapeutic choice for various malignancies, including HCC. ICIs activate T cells in multiple ways, and they may assist in reversing the exhausted phenotype of tumor-infiltrating lymphocytes [8]. As a starting point, this review will explore the fundamental ideas of physiological hepatic immunogenic tolerance and the pathological evasive procedure of anti-tumor immunity in HCC, followed by clinical studies involving immune checkpoint agent monotherapy and combination therapies. Finally, both obstacles and future instructions in this field will be discussed.

## Immune checkpoints in HCC

The liver is an entire structure with a complex and dynamic immune system that functions as an initial line of host protection against antigens and microbial products from the intestines and systemic circulation without inducing unwanted immune responses. According to reports, the hepatic immune system is “immunologically tolerogenic,” which might be harmful in the case of pathological situations. Patients with underlying inflammation caused by hepatitis B and C viruses, fibrosis, and cirrhosis are prone to developing and eventually causing HCC. This physiologic environment can subvert inflammation and tumor development [9]. Immunotherapy is a new therapeutic approach that can be a successful therapy for HCC because it is an inflammation-related malignancy at the first level, which makes it more likely to be efficacious. Second, since the liver is an immune-privileged organ, immunotherapeutic agents are not processed and have predictable pharmacokinetics in individuals with cirrhosis [10]. Third, the liver is immunologically tolerogenic to antigens, balanced by naive T-cell stimulation and various immunosuppressive pathways, such as dysregulation of cytokine release, antigen and immune checkpoint expression, and alterations in the local immune microenvironment [11, 12].

Different immune cells are infiltrated in the HCC TME, particularly myeloid cells, natural killer cells, and T-cells. Since most HCC patients have chronic inflammation and cirrhosis, the tumor environment becomes complex, impacting the tumor behavior and treatment results. Immune cells and tumors interact in a complex fashion, giving rise to the exhaustion of pro-inflammatory cells and the over-dominance of regulatory leukocytes that impedes anti-tumor immunity, mediated by cytokines and signaling pathways [13, 14]. According to Yu et al., increased immune infiltration enhanced overall survival. The study evaluated distinct immunological clusters depending on their predictive value, revealing that cell populations with high T-cells and low levels of macrophages were associated with better outcomes. It was observed that a subgroup of tumor-associated macrophages (TAMs) (M1) is linked to improved results. Poor prognosis is linked to the accumulation of myeloid-derived suppressor cells (MDSCs), TAMs, CD4+/CD25+/FOXP3+ immune-suppressive T-cells (Treg), exhausted Th1 CD4+, CD8+ T-cells, dysfunctional NK cells and the expansion of Th2 CD4+ T-cells [15]. Immune checkpoint molecules like PD-1, CD274 (PD-L1), CTLA-4, and LAG-3 were detected in groups with high levels of CD8+ T-cells. However, these gatherings were linked to a poor prognosis, leading researchers to suggest that these molecules are involved in HCC immune exhaustion [16].

**Fig. 1:**
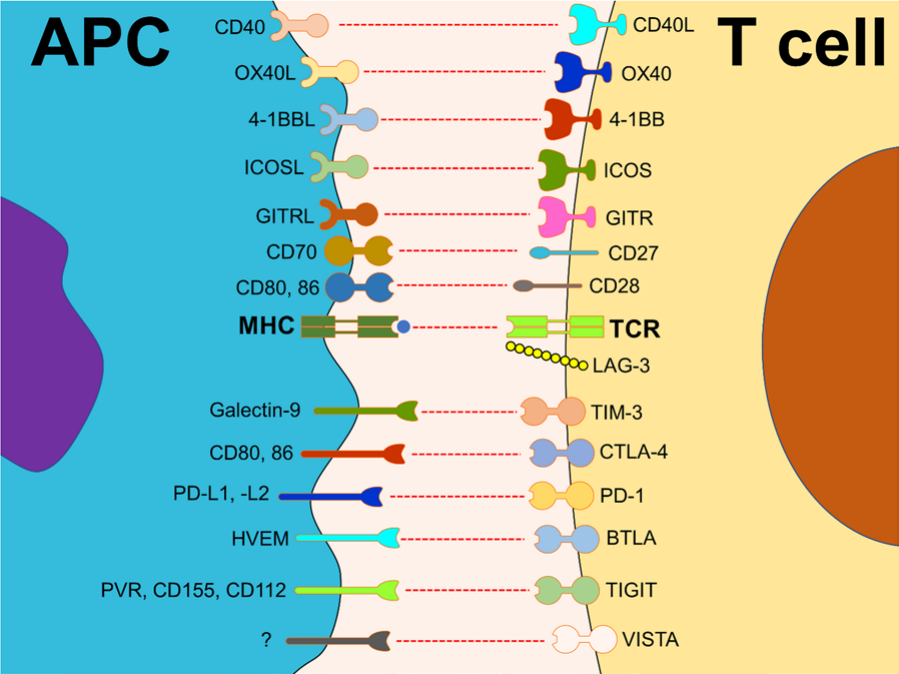
Main co-stimulatory and inhibitory immune checkpoints. Immune checkpoints are a set of various receptors and ligands that play a critical role in immune system regulation, including co-stimulatory and inhibitory molecules

In contrast to traditional cytotoxic medicines, immune checkpoint inhibitors help the host immune system fight cancer. Immune checkpoints maintain a balance between pro-inflammatory and anti-inflammatory signals under homeostatic settings. These immunological checkpoints are a collection of inhibitory and stimulatory mechanisms that influence immune cell function directly. Malignant cells disturb this equilibrium by inducing an immunosuppressive condition that promotes immune evasion and tumor progression [17]. Cancer cells attract Tregs, decrease tumor antigen expression, promote T cell tolerance and/or death, and secrete immune suppressive cytokines that activate inhibitory immunological checkpoints. As a result, the TME becomes distinct and immunosuppressive. Immune checkpoint inhibitors work by blocking the effects of specific inhibitory pathways to overcome immunosuppressive circumstances [18, 19]. The introduction of ICIs, a therapeutic class of monoclonal antibodies that target immune checkpoints, has recently changed the systemic therapy of HCC. As a result, administering ICIs to these HCC patients is expected to be advantageous [20]. ICIs have shown promise in the fight against HCC. In 15–20% of patients, they result in objective remissions that are long-lasting and linked to prolonged survival [21]. Since ICIs have been a cornerstone of treatment for various cancers, multiple clinical trials have been undertaken. Others are ongoing to evaluate their safety and effectiveness in various solid and hematological malignancies [22]. The primary immune checkpoints are CTLA-4, PD-1, TIM-3, VISTA, and LAG-3 (Fig. 1).

### PD-1/PD-L1

Ishida et al. first characterized PD-1, a then-novel member of the immunoglobulin gene group, on murine immune cells in 1992 and demonstrated that PD-1 could produce a classical type of programmed cell death. Researchers discovered that PD-1-knockout mice experienced lupus-like autoimmune disorders in 1999, indicating that PD-1 functions as an immunological gatekeeper. Nivolumab, the first anti-PD-1 monoclonal antibody, was approved by the Food and Drug Administration (FDA) in 2014 for second-line therapy of unresectable or metastatic melanoma. Since then, the FDA has approved numerous more anti-PD-1/L1 antibodies for cancer treatment [23]. PD-1 ligand (PD-L1; CD279 and B7-H1) is a 33-kDa type 1 transmembrane glycoprotein with 290 amino acids and Ig- and IgC domains in its extracellular region that belongs to the B7 class of immunological checkpoints [24]. The PD-1/PD-L1 pathway regulates immunological tolerance induction and maintenance inside the TME. In cancer, degeneration of anti-tumor immune responses is caused by the activation, proliferation, and cytotoxic secretion of T cells in response to the activity of PD-1 and its ligand PD-L1 or PD-L2. PD-L1 is linked to an immunological milieu rich in CD8 T cells, the generation of Th1 cytokines and chemical factors, interferons, and particular gene expression [25]. This pathway serves a physiological function in self-tolerance and suppressing immune responses to immunological responses. Honjo’s group discovered PD-L1 as a PD-1 ligand in 2000, as a receptor produced by antigen-presenting cells (APCs), mainly in the heart, pulmonary, renal, and placenta systems [26]. PD-L1 expression is increased in activated hematopoietic cells, particularly APCs like macrophages and dendritic cells. More significantly, PD-L1 is expressed in many tumor cells and virus-infected cells. When activated by PD-1, it directly suppresses T-cell proliferation and T-cell effector activities such as IFN-gamma production and cytotoxic action against target cells [27]. The expression of PD-1 in CD8+ T cells was enhanced in HCC patients. Generally, PD-L1 is found on hepatocytes, hepatic stellate cells, liver sinusoidal endothelial cells (LSEC), and Kupffer cells, but PD-L2 is found only on dendritic cells [28]. T cell activity in the hepatic TME is inhibited by PD-L1 expression in HCC cells. Unsurprisingly, elevated PD-L1 expression in cancer cells predicted HCC recurrence [29]. M1 macrophages have promoted PD-L1 expression in HCC cells, suggesting M1 macrophages’ pro-tumor activity [30]. The expression of PD-L1 in HCC cells hinders the role of T cells in the hepatic TME. Assessments of HCC resection samples revealed more fabulous expression of PD-L1 and its correlation with tumor aggressiveness and poor prognosis in individuals who were never treated with immunotherapy [31]. Detailed phenotypic flow cytometry investigations of fresh biopsies acquired from progressed HCC patients before anti-PD-1/PD-L1 therapy revealed that responders to anti-PD-1/PD-L1 treatment had a high baseline incidence of PD-1 high CD8+ T cells in malignant cells [32]. This is consistent with a recent study that looked at CD8+ T cells extracted from HCC tissue and found that tumors with a high number of PD-1 high CD8+ T cells are more responsive to PD-1 blocking [33]. Also, significant numbers of PD-1+ intra-tumoral lymphocytes suggest cytokine-induced killer cell survival advantage in HCC patients [34].

### LAG-3

LAG-3 is a member of the immunoglobulin superfamily and a CD4 ancestral homolog created due to gene duplication. As with CD4, LAG3 binds to MHC class II (MHCII), FGL-1, -synuclein fibrils (-syn), and the lectins galectin-3 (Gal-3) and lymph node sinusoidal endothelial cell C-type lectin (LSECtin). LAG3 suppresses host cell activation as an immunological checkpoint and generally increases a more repressive immune response. LAG3, for example, inhibits cytokine and granzyme production and growth in T cells while boosting differentiation into Tregs [35]. Gal-3, a 31-kDa galactose-binding lectin, modulates T cell activation. Immunoprecipitation has been illustrated as associated with LAG-3 and limits interferon-secretion by limiting interferon-secretion CD8+ T cells in vitro, demonstrating that Gal-3 is also a LAG-3 ligand [36]. Gal-3 can be found on the surface of several malignancies, including lung cancer, prostate cancer, colorectal cancer, and breast cancer. As a result, it regulates CD8+ T cells through a variety of methods. It has also been hypothesized that LSECtin is a LAG-3 ligand. LSECtin, a member of the DC-SIGN family of molecules, binds to the four glycosylated sites on LAG-3. LSECtin is expressed in the liver and melanoma tumor cells, implying that LAG-3 modulates CD8+ T and natural killer (NK) cell activity [14]. Fibrinogen-like protein 1 (FGL1) was recognized as a novel LAG3 ligand. FGL1 is a fibrinogen family member with an analogous structure to fibrinogen beta and gamma; however, it has no admitted involvement in platelets or clot formation. Hepatocytes generally release FGL1 in the liver, but tumor cells can also express large amounts of FGL1, corresponding to poor patient prognosis and immunotherapy resistance [37]. Frequently co-expressed with PD-1 on exhausted T cells, LAG-3 has become an exciting target for immune-modulating agents either alone or in combination with inhibitors of the PD-1/PD-L1 axis. Anti-LAG-3 agents are under investigation in phase I–III trials in a wide array of solid tumors, including lung, gastric, head and neck, hepatocellular and renal cancer, and lymphoma and melanoma [38, 39].

### CTLA-4

CTLA-4 is a CD28-related cell surface receptor that binds to CD80 and CD86 [40]. Two consecutive signals are required for T-cell activation. First, antigens presented in setting with the major histocompatibility complex (MHC) I or II on specialized APCs attach to T-cell receptors [41]. The second phase is translating TCR stimulation into T-cell activation. It needs a co-stimulatory signal when B7 molecules on the APC surface interact with CD28 receptors on T cells. The inhibitory molecule CTLA-4 is then expressed on the surface of T cells. CTLA-4 suppresses B7-CD28 binding by interacting with the identical ligands, preventing the co-stimulatory signal and suppressing T-cell activation and proliferation [42, 43]. CTLA-4 can potentially stop T cells from initiating responses by raising the threshold of signals necessary for complete activation and halting ongoing T-cell reactions. This inhibitory signal affects downstream targets of CTLA4, like cytokine generation by Th1 and Th2 cells and essential cell cycle components. As a result, researchers hypothesized that inhibiting the CTLA4-B7 interaction would lead to increased and extended T-cell activation, as evaluated by the higher release of IL-2, IFN-γ, IL-3, IL-4, IL-5, and IL-10, as well as more clinically relevant anti-tumor immune responses [44–46]. Since CTLA-4 has a significant regulatory impact on immune responses, antibodies against either mouse or human CTLA-4 have been established to support immune responses against cancer [47]. In early 1996, Leach et al. reported that administering anti-CTLA-4 antibodies to mice with pre-existing tumors considerably decreased tumor development [48]. Ipilimumab, the first anti-CTLA-4 monoclonal antibody to reach the clinic [49], was approved in 2011 to treat melanoma patients [50]. Tremelimumab was also the first anti-CTLA-4 antibody studied in HCC [51].

### TIM-3

TIM-3, also recognized as HAVCR2, is a member of the TIM gene family. In humans, the TIM family consists of TIM-1, TIM-3, and TIM-4 and is found on chromosome 5q33.2. This family, which contains TIM-1 through TIM-8 in mice, is located on chromosome 11B1.1 [52]. Galectin-9 (Gal-9), carcinoembryonic antigen cell adhesion molecule 1 (CEACAM-1), high-mobility group protein B1 (HMGB1), and phosphatidylserine (PS) are all TIM-3 ligands. This carbohydrate-binding protein recognizes the N-linked sugar chain structure in the TIM-3 immunoglobulin (IgV) domain. TIM-3/Gal-9 can suppress cancer immunity by suppressing T-cell immunity. The interaction of the TIM-3 IgV domain with Gal-9 can result in the termination of T helper 1 (Th1) immunological responses [53]. TIM3 has received the most attention within the TIM family because of its role in the modulation of immune responses in autoimmune disorders and cancer. While it was initially discovered as a molecule expressed by interferon-producing CD4+ and CD8+ T cells, TIM3 has been represented by various other cell types, including Treg cells and myeloid cells, NK cells, and mast cells [54]. Several studies indicate that the addition of cytokines such as IL-4, TGF-, and IL-6 in the TME induces the production of TIM-3 in HCC cells [55]. The expression of TIM-3 in HCC cells promotes tumor development via IL-6 autosecretion, which also boosts HCC cell metastatic capacity by increasing epithelial–mesenchymal transition (EMT) [56]. Wu et al. discover that TIM-3 expression on CD4+ and CD8+ T cells increases through chronic hepatitis, resulting in lower IFN- and T-bet mRNA plasma levels in these cells. Furthermore, the degree of TIM-3 expression is linked with illness severity and is favorably connected with alanine aminotransferase (ALT), aspartate aminotransferase (AST), the international normalized ratio (INR), and total bilirubin (TB) [57]. Furthermore, mounting data suggests that TIM-3 may have a central role on TILs, leading to immunosuppression in HCC TME. As a result, Yan et al. discovered higher TIM-3 expression levels on CD4+ and CD8+ T cells from HCC patients’ peripheral blood. Additionally, it has been shown that the expression of TIM-3 is higher on CD4+ and CD8+ T lymphocytes entering tumor tissues than on cells infiltrating surrounding tissues [58]. The importance of NF-κB in sustaining the M2 phenotype and protumoral activity of TAMs in many malignancies, including HCC, has recently been highlighted. It has been established that TIM-3 mediates NF-B pathway activation in TGF-β or IL-4-stimulated macrophages [59]. Moreover, tumor-infiltrated conventional NK (cNK) and liver resident NK (LrNK) cells were shown to have increased TIM-3 expression in Patients with hcc, which decreases cytokine release and cytotoxicity since TIM-3 impairs the downstream Akt/mTORC1 pathway in NK cells [60].

### TIGIT

TIGIT was found as a new member of the Ig superfamily in 2009 [61]. It is an inhibitory receptor found on immune cells such as effector and memory T cells and Treg cells, follicular T cells, and NK cells [53, 62, 63]. An immunoreceptor tyrosine-based inhibitory motif (ITIM) and an immunoglobulin tail tyrosine (ITT)-like motif are found in the cytoplasmic tail and are highly conserved between mice and humans [64]. Numerous possible mechanisms for TIGIT-dependant suppression of effector T and NK cells have been suggested. TIGIT can act either cell-extrinsically as a CD155 ligand or cell-intrinsically by interfering with DNAM-1 co-stimulation or directly providing repressive signals to the effector cell [61, 64, 65]. It is unknown whether all these processes are active in every TIGIT-expressing cell or if TIGIT action varies between CD4+ T cells, CD8+ T cells, and NK cells. TIGIT also increases Treg suppressive capabilities when expressed on Tregs, potentially inhibiting various immune cells [63, 66]. In mouse pre-clinical models and cancer patients, TIGIT expression on tumor-infiltrating CD8+ T cells often links with augmented expression of other inhibitory receptors like PD-1, LAG-3, TIM-3, and decreased expression of DNAM-1 [65, 67, 68]. So, TIGIT marks dysfunctional CD8+ T cells with reduced cytokine secretion and degranulation abilities [69, 70]. According to a recent study, gastric cancer cells suppress CD8 T-cell metabolism via CD155/TIGIT signaling, which blocks CD8 T-cell effector activities, leading to hyporesponsive anti-tumor immune reaction [71]. Furthermore, the expression of TIGIT and its ligand, CD155, was investigated in the cancerous tissues of HCC patients. The findings suggested that the TIGIT/CD155 signaling pathway could be a promising target for diagnosing and treating HCC [72].

### VISTA

VISTA was introduced as an immune checkpoint receptor in 2011 and is expressed on tumor-infiltrating lymphocytes (TILs) and a range of immune cells such as macrophages and T cells. VISTA supports inhibiting T cell stimulation, proliferation, and cytokine release [73]. VISTA differs from PD-1 and CTLA4 in that it can operate as both a ligand expressed on APCs and a receptor on T cells [74, 75]. Most research has documented VISTA’s immune-suppressive function and the potential of VISTA deficiency or anti-VISTA therapy to elevate immune reactions [76]. Regarding human malignancies and murine models, VISTA is mainly expressed on immune cells in the TME. Still, it has also been detected on tumor cells in human lung, kidney, colorectal, endometrial, and ovarian cancers, among others [77–80]. Although several studies have highlighted that VISTA expression is associated with tumor progression, some other investigations have indicated that VISTA expression by TILs is associated with better patient survival [81, 82]. HCC is linked to chronic inflammation as well as inhibitory immunological responses. VISTA is a novel negative checkpoint regulator, but its expression is substantially related to CD8+ TILs; hence dual positive VISTA+/CD8+ revealed a favorable TME, resulting in a better OS than VISTA-negative expression in 183 HCC tissue microarray [83, 84].

## Immune checkpoint inhibitor monotherapy for HCC

**Fig. 2:**
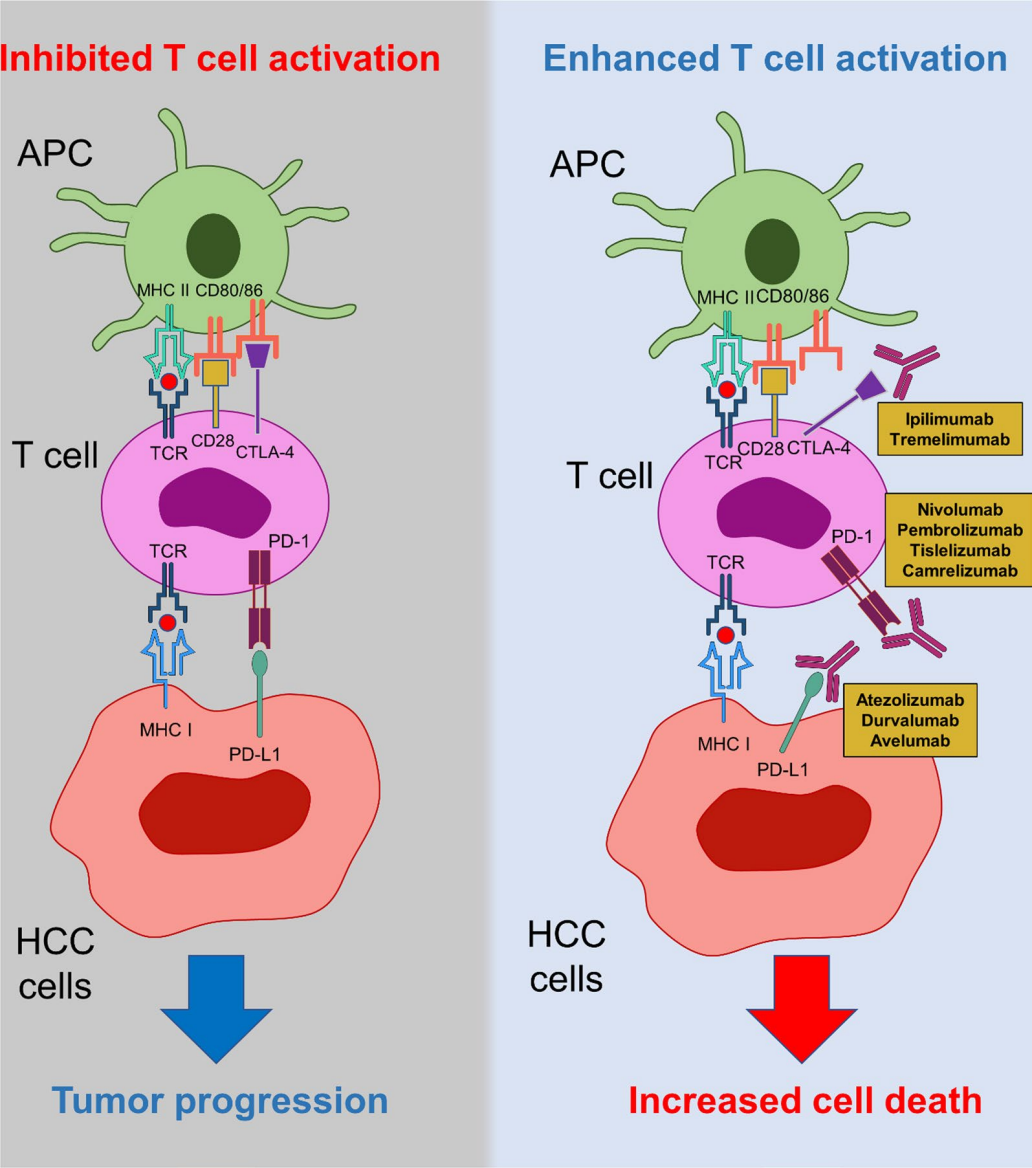
Difference between inhibited and active immune checkpoint conditions. Using monoclonal antibodies against immune checkpoints such as CTLA-4 and PD-1/PD-L1 could barricade the inhibited activity of T cells against tumor cells. These inhibitions could significantly increase cell death and induction of apoptosis. In contrast, the interaction of immune checkpoints and their ligands suppress a proper anti-tumor immune response

Monoclonal antibodies capable of blocking immune checkpoint molecules have demonstrated anti-tumor function against many human cancers (Fig. 2). Immunotherapy with checkpoint blockade agents, alone or in combination, has encouraged preliminary outcomes in patients with HCC (Table 1).

**Table 1:** Clinical trials conducted with ICIs (monotherapy and combination therapy) in HCC

Target	Drug	epitope	Intervention	Phase	Status	Trial ID	Refs.
Monotherapy							
PD-1	Nivolumab	PD-1N-loop	–	III	Active, not recruiting	NCT03383458	[104]
PD-1	Pembrolizumab	PD-1 CD loop	–	II	Active, not recruiting	NCT02702414	[105]
PD-1	Pembrolizumab	PD-1 CD loop	–	III	Completed	NCT02702401	[106]
PD-1	Pembrolizumab	PD-1 CD loop	–	III	Active, not recruiting	NCT03062358	[107]
PD-1	Tislelizumab	PD-1 CC’ loop	–	I	Completed	NCT02407990	[108]
PD-L1	Durvalumab	PD-1 C, F, G, CC’ loop and N-terminal	–	II	Completed	NCT01693562	[109]
PD-L1	Avelumab	C, C′, F, G and CC′ loop of PD-L1	–	II	Completed	NCT03389126	[110]
Combination therapy							
PD-L1	Atezolizumab	PD-L1 CC′FG antiparallel β-sheet and the BC, CC′, C′C″, and FG loops	Bevacizumab	I	Completed	NCT02715531	[111]
PD-L1	Atezolizumab	PD-L1 CC′FG antiparallel β-sheet and the BC, CC′, C′C″, and FG loops	Cabozantinib	I, II	Recruiting	NCT03170960	[112]
PD-1	Nivolumab	PD-1N-loop	Cyclophosphamide IRX-2	I	Recruiting	NCT03655002	[113]
PD-1	Nivolumab	PD-1N-loop	Fluorouracil Recombinant Interferon Alpha 2b-like Protein	I, II	Recruiting	NCT04380545	[114]
PD-1	Sintilimab	FG loop on PD-1	Lenvatinib TACE-HAIC	II	Recruiting	NCT04814043	[115]
PD-1, PD-L1	Pembrolizumab Nivolumab Atezolizumab Avelumab Durvalumab	PD-1 CD loop PD-1N-loop, PD-1 C, F, G, CC’ loop and N-terminal, PD-L1 CC′FG antiparallel β-sheet and the BC, CC′, C′C″, and FG loops	N-803	II	Active, not recruiting	NCT03228667	[116]
PD-1	Nivolumab	PD-1N-loop	Radiofrequency Ablation	IV	Completed	NCT04707547	[117]
PD-1	Nivolumab	PD-1N-loop	Regorafenib	II	Recruiting	NCT04310709	[118]
PD-1, CTLA-4	Durvalumab Tremelimumab	PD-1 C, F, G, CC’ loop and N-terminal	SBRT	II	Recruiting	NCT04988945	[119]
CTLA-4	Tremelimumab	CDR loops	Sorafenib	II	Active, not recruiting	NCT01008358	[85]
PD-1	Nivolumab	PD-1N-loop	Sorafenib	II	Active, not recruiting	NCT01658878	[120]
PD-1	Nivolumab	PD-1N-loop	Sorafenib	III	Active, not recruiting	NCT02576509	[121]
PD-1	Tislelizumab	PD-1 CCʹ loop	Sorafenib	III	Active, not recruiting	NCT03412773	[122]
PD-1	Anti-PD-1	Not mentioned	TACE-HAIC	II	Recruiting	NCT04814030	[123]

Tremelimumab is a monoclonal antibody that inhibits CTLA-4, a co-receptor that inhibits T cell activation and proliferation. A phase I study of tremelimumab in patients with HCC was recently published (NCT01008358). The occurrence of chronic hepatitis C virus (HCV) infection in HCC patients presented an exceptional opportunity to investigate tremelimumab’s anti-tumor and antiviral effects at the same time. There was a good safety profile, and no patient required steroids due to significant immune-mediated side effects. The disease control rate was 76.4%, with a partial response rate of 17.6%. There was a considerable reduction in viral load, and this antiviral impact was linked to an enhanced specific anti-HCV immune response. The findings of the first trial indicated that this CTLA-4-blocking drug could have both antitumoral and antiviral effects against HCV infection [51].

Nivolumab is a monoclonal antibody that recognizes and inhibits the PD-1 immune checkpoint. It is made entirely of human immunoglobulin G4, restoring the previously inhibited anticancer function of effector T cells. The safety and efficacy of nivolumab as a monotherapy (CheckMate 040) were assessed in a multicenter, non-comparative, open-label phase 1/2 study in patients with HCC with or without chronic viral hepatitis (HCV or HBV). Nivolumab was shown to have a manageable safety profile, with no new signals seen in HCC patients. Long-term objective responses demonstrate Nivolumab’s ability to treat HCC. Based on the outcomes of this research, the Food and Drug Administration (FDA) hastened the approval of nivolumab for treating patients with HCC who had formerly been treated with Sorafenib in the United States [85, 86]. A phase III randomized investigation (NCT02576509, CheckMate-459) of nivolumab monotherapy compared with Sorafenib in the first-line setting is underway. Another phase III trial (CheckMate 9DX, NCT03383458) examined whether nivolumab can enhance recurrence-free survival in HCC patients who have had complete resection or obtained a complete response following local ablation and are at high risk of recurrence when compared to placebo.

In numerous malignancies, including HCC, pembrolizumab, an anti-PD-1 monoclonal antibody, has shown anti-tumor effectiveness and a controllable toxicity profile. A phase II trial assessed the efficacy and safety of pembrolizumab as monotherapy in individuals with HCC (KEYNOTE-224, NCT02702414). In HCC patients who had previously been treated with Sorafenib, pembrolizumab medication resulted in long-lasting responses and improved progression-free and overall survival [87]. A phase III trial evaluating patients with either a pembrolizumab or a placebo group (KEYNOTE-240, NCT02702401) is currently underway and compares pembrolizumab to best supportive care in HCC patients. In addition, a trial of pembrolizumab or placebo in conjunction with optimal supportive care in Asian patients with previously treated HCC is now being conducted continuously (NCT03062358).

The investigational IgG4 anti-PD-1 mAb tislelizumab (BGB-A317) was engineered to attach slightly to FcR on macrophages; its unique pharmacodynamic properties allow the drug to avoid antibody-dependent phagocytosis, which is a possible mechanism of anti-PD-1 therapy resistance [62]. The HCC cohort of a phase I trial with tislelizumab had a 12.2% overall response rate and a 51.0% disease control rate; the most prevalent treatment-emergent side effects were reduced appetite, rash, weight loss, and cough [63]. A phase III trial (RATIONALE 301, NCT03412773) comparing tislelizumab versus Sorafenib in the first-line treatment of HCC is being conducted due to the preliminary safety profile of anti-tumor activity shown in HCC patients.

Inflammatory response to PD-L1 is blocked by durvalumab, a human immunoglobulin G1 monoclonal antibody. A phase I/II trial of durvalumab monotherapy for solid tumors, including HCC, was completed, with a 10% response rate and a median survival duration of 13.2 months for 40 HCC patients. In addition, Durvalumab exhibited promising anticancer efficacy and overall survival in HCC patients [88]. Another human IgG1 mAb, avelumab, is now being investigated in phase II research in patients with advanced HCC who have previously received sorafenib treatment (NCT03389126). Ultimately, atezolizumab is a humanized IgG mAb that targets PD-L1. In patients with advanced HCC, the phase Ib research GO30140 compared atezolizumab monotherapy to a combination of atezolizumab and the anti-VEGF bevacizumab. The monotherapy arm had a median progression-free survival of 3.4 months, compared to 5.6 months in the combination arm [89].

Furthermore, HCC treatment may target TIM-3 and LAG-3 immunological checkpoints. When macrophages were depleted of TIM-3, they prevented tumor growth in vitro and in vivo in HCC patients, as observed by Yan et al. [59]. In addition, LAG-3 has been determined to have an abnormal expression in a wide range of human malignancies, including HCC [90]. As a result, targeting TIM-3 and LAG-3 in the therapy of HCC could be beneficial.

These encouraging findings suggest that checkpoint inhibitors could be used in combination. In addition, it has been proposed that the anticancer response to ICIs might be improved if these drugs were to be administered in combination with other treatments. Therefore, in the following section, we will present several combined therapies effective against HCC.

## Combination of ICI with other therapeutic approaches

### ICI and chemotherapy

When chemotherapy and ICIs are used simultaneously, they have a synergistic effect: by destroying tumor cells, chemotherapy causes the release of cancer antigens, which improves cancer antigen presentation by APCs. Furthermore, chemotherapy has an immunomodulatory effect, boosting and suppressing the immune system. While it increases the number of CD8+ T cells infiltrating the TME and enhances the maturation and activity of APCs, it also increases the downregulation of myeloid-derived suppressor cells (MDSCs) and Tregs production, as well as TME infiltration [91]. A case report study on patients with gastrointestinal cancers, including HCC, implies that preservation targeted therapy or chemotherapy may be more effective after immunotherapy. Furthermore, earlier initiation of immunotherapy could positively regulate responses to outcomes with other systemic treatment options. Surprisingly, Initially unresponsive to first-line targeted therapy for HCC, two patients in this case series demonstrated a surprise response to salvage targeted therapy after exposure to immunotherapy, providing credibility to the idea of a potential alteration in the TME following interim ICI exposure [92]. According to current clinical trials, not only will combinational therapy or innovative molecular targeted therapy transform systemic therapy for patients with advanced HCC, but the coming medicines or techniques will significantly alter the treatment of early-stage and intermediate-stage HCC. A first generally accepted adjuvant therapy for early-stage HCC could be an ICI or an anti-angiogenic medication with minimal toxicity. TACE's efficacy for patients with intermediate-stage HCC will also be enhanced by combining ICIs and an anti-angiogenic drug [93].

### ICI and radiotherapy (RT)

Radiation might affect the co-stimulatory and inhibitory signals elicited by ICI. Based on this evidence, the combination of RT with ICIs is considered synergistic, and preliminary evidence suggests its use in treating HCC [94]. It has been proven that RT induces PD-L1 expression in tumor cells and that anti-PD-L1 medicines have a potential therapeutic impact against HCC. Furthermore, compared to RT alone or the anti-PD-L1 agent alone, the combination of an anti-PD-L1 drug plus RT dramatically boosted cytotoxicity and CD8 T-cell proliferation [95]. Furthermore, RT increased TIM-3 expression in HCC cell lines, and the combination of an anti-TIM-3 drug and radiation increased cytotoxic effects and CD8 T-cell proliferation.

Additionally, compared to radiation or an anti-TIM-3 drug alone, the combination of an anti-TIM-3 agent with radiation substantially inhibited tumor development. Furthermore, in line with the tumor growth statistics, the combination group displayed a considerable increase in survival [96]. Therefore, more emphasis should be paid to combining different ICI and RT to improve the efficacy of therapeutic methods in HCC therapy.

## Resistance to ICI and overcoming

Both primary and acquired ICI resistance exist, with the former describing those patients who respond to treatment initially but then experience clinical or radiologic progression afterward. The latter refers to those patients who initially respond to treatment but then experience clinical or radiologic progression afterward. Much work has been expended on combination methods, frequently in conjunction with empiric orthogonal therapies, to address the difficulty of primary resistance to extending the receptive population [97]. They identified antigens on major histocompatibility complexes (MHCs) of APCs. MHC class I antigen presentation to tumors is mediated by the synchronized expression of several genes. One crucial gene, beta-2-microglobulin (B2M), is responsible for the cell surface stability of MHC class 1 molecule and the facilitation of peptide loading [98]. In recent years, one of the few reoccurring results in acquired resistance to ICIs has been truncating B2M mutations. In the study of Zaretsky et al., which analyzed data from four melanoma patients who had established immunotherapy resistance, another patient exhibited a homozygous acquired truncating B2M mutation [99]. Despite being among the most prevalent recurrent findings in acquired resistance, biallelic B2M mutations appear rare at baseline. They are not a well-established mechanism of ICI primary resistance.

Furthermore, IFN-γ released by effector T cells initiates a signaling cascade in tumor cells via the JAK-STAT pathway, promoting MHC class I and PD-L1 production and thus can promote tumor cell death in a variety of ways. Stimulating receptor-associated kinases JAK1 and JAK2 by IFN-γ binding to the heterodimeric IFNGR1/IFNGR2 is one of the vital early stages in this pathway. Recent clinical studies have identified JAK1 or JAK2 inactivating mutations, which may cause ICI development [97]. Neoantigen-specific T cells may play an essential part in the functional response to ICIs. As a result, loss of somatic mutations encoding potential tumor-specific neoantigens via clonal expansion, epigenetic suppression, or copy number reduction may result in immune escape and clinical progression [100]. In pre-clinical models, deletion of the tumor suppressor PTEN, which is essential in regulating PI3K function, promotes immunosuppressive cytokines and decreases T cell effector IFN-γ, inhibiting T-cell driven infiltration and immunity [101]. WNT/βcatenin activation, like PTEN loss, has been associated with the generation of immunosuppressive cytokines, changes in dendritic cell priming, activation of Tregs, and a lack of substantial T cell infiltration in melanoma, suggesting the role of β-catenin in ICI resistance [102]. Several clinical trials utilizing immunotherapeutic medicines in conjunction with targeted drugs, cytotoxic chemotherapy, and/or radiation are now underway to give long-term disease management to more patients. Combination treatments for overcoming innate resistance by attacking putative immune evasion pathways inside the TME are in different stages of research. They show promise to personalize cancer immunotherapy and perhaps improve immunological memory [103]. Biomarkers for ICI resistance and response are currently being investigated, as are novel immune modulatory medications and innovative combinations of immune modulators and ICI with other cancer therapies in the early stages of clinical trials.

## Future prospective and conclusion

In recent years, ICIs have been revealed to have a successful therapeutic result on HCC patients, with a disease control rate of around 20% [85, 87]. However, identifying and treating irAEs in HCC patients may provide unique challenges because of the liver’s distinct hepatic immunobiology and chronic inflammatory illnesses, such as cirrhosis and viral hepatitis, that affect the liver [124]. Given that immune checkpoint inhibition fails to help many patients with HCC, and few predictive indicators have been identified to select people with HCC who may benefit, ICIs should be examined further in HCC treatment. Regarding the adverse effect of ICI as a therapeutic approach (Table 2), different methods are now being investigated to enhance the efficacy of ICIs and improve patient selection for treatment [125].

**Table 2:** Side effects of immune checkpoint inhibitors

Organ	Immune-related adverse effects (irAEs)
Gastrointestinal tract	Nausea, vomiting, dysphagia, epigastric pain, abdominal pain, hematochezia, and diarrhea
Kidney	Acute interstitial nephritis
Endocrine	Hypophysitis, thyroid dysfunction, primary adrenal insufficiency, hypoparathyroidism, and type 1 diabetes mellitus
Hematologic complications	anemia, thrombocytopenia, leukopenia, and neutropenia
Joint	joint swelling, warmth, erythema, and joint pain
Skin	Rash, pruritus, and vitiligo
Nervous system	Inflammatory (encephalitis, myelitis, vasculitis, and meningitis) and peripheral neuromuscular autoimmune disorders (myasthenia gravis and Guillain–Barre syndrome)
Ocular system	Uveitis
Heart	Myocarditis, arrhythmias, conduction disease, acute coronary syndrome, congestive heart failure, and pericardial disease
Lung	Pneumonitis

Given that numerous immunosuppressive pathways support HCC-mediated immune tolerance in an intrinsically tolerogenic liver environment, it is reasonable to suppose that dual or triple immunotherapeutic combinations could be the most profitable therapeutic development strategies for HCC [126].

Clinical trials analyzing mono- or combination therapies, including ICIs, are ongoing in HCC. Also, investigations on the pathways involved in combination strategies and the recognition of predictive biomarkers of response and irAEs continue to enhance clinical advantage and stop improper therapies, consequently diminishing probably enhanced toxicities, given the coexisting liver dysfunction in HCC patients [127]. The findings of these studies will make it possible to treat patients with HCC more safely and effectively by implementing more personalized immunotherapy. To conclude, immunotherapy in HCC is both promising and challenging.

## Data Availability

Not applicable.

## References

[1] Sia D, Villanueva A, Friedman SL, Llovet JM (2017). Liver cancer cell of origin, molecular class, and effects on patient prognosis. Gastroenterology.

[2] Schlachterman A, Craft WW, Hilgenfeldt E, Mitra A, Cabrera R (2015). Current and future treatments for hepatocellular carcinoma. World J Gastroenterol.

[3] Rapisarda V, Loreto C, Malaguarnera M, Ardiri A, Proiti M, Rigano G, Frazzetto E, Ruggeri MI, Malaguarnera G, Bertino N (2016). Hepatocellular carcinoma and the risk of occupational exposure. World J Hepatol.

[4] Yang JD, Hainaut P, Gores GJ, Amadou A, Plymoth A, Roberts LR (2019). A global view of hepatocellular carcinoma: trends, risk, prevention and management. Nat Rev Gastroenterol Hepatol.

[5] Eggert T, Greten TF (2017). Tumor regulation of the tissue environment in the liver. Pharmacol Ther.

[6] Leone P, Solimando AG, Fasano R, Argentiero A, Malerba E, Buonavoglia A, Lupo LG, De Re V, Silvestris N, Racanelli V (2021). The evolving role of immune checkpoint inhibitors in hepatocellular carcinoma treatment. Vaccines.

[7] Sangro B, Chan SL, Meyer T, Reig M, El-Khoueiry A, Galle PR (2020). Diagnosis and management of toxicities of immune checkpoint inhibitors in hepatocellular carcinoma. J Hepatol.

[8] Liu X, Qin S (2019). Immune checkpoint inhibitors in hepatocellular carcinoma: opportunities and challenges. Oncologist.

[9] Siu EH-L, Chan AW-H, Chong CC-N, Chan SL, Lo K-W, Cheung ST (2018). Treatment of advanced hepatocellular carcinoma: immunotherapy from checkpoint blockade to potential of cellular treatment. Transl Gastroenterol Hepatol.

[10] Hato T, Goyal L, Greten TF, Duda DG, Zhu AX (2014). Immune checkpoint blockade in hepatocellular carcinoma: current progress and future directions. Hepatology.

[11] Nemeth E, Baird AW, O’Farrelly C. Microanatomy of the liver immune system. In: Seminars in immunopathology. Springer; 2009:333–43. 10.1007/s00281-009-0173-4 19639317

[12] Thomson AW, Knolle PA (2010). Antigen-presenting cell function in the tolerogenic liver environment. Nat Rev Immunol.

[13] Abd El Aziz MA, Facciorusso A, Nayfeh T, Saadi S, Elnaggar M, Cotsoglou C, Sacco R (2020). Immune checkpoint inhibitors for unresectable hepatocellular carcinoma. Vaccines.

[14] Safarzadeh E, Asadzadeh Z, Safaei S, Hatefi A, Derakhshani A, Giovannelli F, Brunetti O, Silvestris N, Baradaran B (2020). MicroRNAs and lncRNAs—a new layer of myeloid-derived suppressor cells regulation. Front Immunol.

[15] Yu S, Wang Y, Hou J, Li W, Wang X, Xiang L, Tan D, Wang W, Jiang L, Claret FX (2020). Tumor-infiltrating immune cells in hepatocellular carcinoma: Tregs is correlated with poor overall survival. PLoS ONE.

[16] Pinato DJ, Guerra N, Fessas P, Murphy R, Mineo T, Mauri FA, Mukherjee SK, Thursz M, Wong CN, Sharma R (2020). Immune-based therapies for hepatocellular carcinoma. Oncogene.

[17] Chen L, Flies DB (2013). Molecular mechanisms of T cell co-stimulation and co-inhibition. Nat Rev Immunol.

[18] Vinay DS, Ryan EP, Pawelec G, Talib WH, Stagg J, Elkord E, Lichtor T, Decker WK, Whelan RL, Kumara HS. Immune evasion in cancer: mechanistic basis and therapeutic strategies. In: Seminars in cancer biology. Elsevier; 2015:S185–S98. 10.1016/j.semcancer.2015.03.004 25818339

[19] Pardoll DM (2012). The blockade of immune checkpoints in cancer immunotherapy. Nat Rev Cancer.

[20] Salmaninejad A, Valilou SF, Shabgah AG, Aslani S, Alimardani M, Pasdar A, Sahebkar A (2019). PD-1/PD-L1 pathway: basic biology and role in cancer immunotherapy. J Cell Physiol.

[21] Langhans B, Nischalke HD, Krämer B, Dold L, Lutz P, Mohr R, Vogt A, Toma M, Eis-Hübinger AM, Nattermann J (2019). Role of regulatory T cells and checkpoint inhibition in hepatocellular carcinoma. Cancer Immunol Immunother.

[22] Zhu AX, Kang Y-K, Yen C-J, Finn RS, Galle PR, Llovet JM, Assenat E, Brandi G, Pracht M, Lim HY (2019). Ramucirumab after Sorafenib in patients with advanced hepatocellular carcinoma and increased α-fetoprotein concentrations (REACH-2): a randomised, double-blind, placebo-controlled, phase 3 trial. Lancet Oncol.

[23] Carlino MS, Larkin J, Long GV (2021). Immune checkpoint inhibitors in melanoma. The Lancet.

[24] Sanmamed MF, Chen L (2014). Inducible expression of B7–H1 (PD-L1) and its selective role in tumor site immune modulation. Cancer J (Sudbury, Mass).

[25] Ji M, Liu Y, Li Q, Li X-D, Zhao W-Q, Zhang H, Zhang X, Jiang J-T, Wu C-P (2015). PD-1/PD-L1 pathway in non-small-cell lung cancer and its relation with EGFR mutation. J Transl Med.

[26] Freeman GJ, Long AJ, Iwai Y, Bourque K, Chernova T, Nishimura H, Fitz LJ, Malenkovich N, Okazaki T, Byrne MC (2000). Engagement of the PD-1 immunoinhibitory receptor by a novel B7 family member leads to negative regulation of lymphocyte activation. J Exp Med.

[27] Iwai Y, Hamanishi J, Chamoto K, Honjo T (2017). Cancer immunotherapies targeting the PD-1 signaling pathway. J Biomed Sci.

[28] Mocan T, Sparchez Z, Craciun R, Bora C, Leucuta D (2019). Programmed cell death protein-1 (PD-1)/programmed death-ligand-1 (PD-L1) axis in hepatocellular carcinoma: prognostic and therapeutic perspectives. Clin Transl Oncol.

[29] Gao Q, Wang X-Y, Qiu S-J, Yamato I, Sho M, Nakajima Y, Zhou J, Li B-Z, Shi Y-H, Xiao Y-S (2009). Overexpression of PD-L1 significantly associates with tumor aggressiveness and postoperative recurrence in human hepatocellular carcinoma. Clin Cancer Res.

[30] Zong Z, Zou J, Mao R, Ma C, Li N, Wang J, Wang X, Zhou H, Zhang L, Shi Y (2019). M1 macrophages induce PD-L1 expression in hepatocellular carcinoma cells through IL-1β signaling. Front Immunol.

[31] Macek Jilkova Z, Aspord C, Decaens T (2019). Predictive factors for response to PD-1/PD-L1 checkpoint inhibition in the field of hepatocellular carcinoma: current status and challenges. Cancers.

[32] Jilkova ZM, Aspord C, Kurma K, Granon A, Sengel C, Sturm N, Marche PN, Decaens T (2019). Immunologic features of patients with advanced hepatocellular carcinoma before and during Sorafenib or anti-programmed death-1/programmed death-L1 treatment. Clin Transl Gastroenterol.

[33] Kim H-D, Song G-W, Park S, Jung MK, Kim MH, Kang HJ, Yoo C, Yi K, Kim KH, Eo S (2018). Association between expression level of PD1 by tumor-infiltrating CD8+ T cells and features of hepatocellular carcinoma. Gastroenterology.

[34] Chang B, Shen L, Wang K, Jin J, Huang T, Chen Q, Li W, Wu P (2018). High number of PD-1 positive intratumoural lymphocytes predicts survival benefit of cytokine-induced killer cells for hepatocellular carcinoma patients. Liver Int.

[35] Durham NM, Nirschl CJ, Jackson CM, Elias J, Kochel CM, Anders RA, Drake CG (2014). Lymphocyte Activation Gene 3 (LAG-3) modulates the ability of CD4 T-cells to be suppressed in vivo. PLoS ONE.

[36] Kouo T, Huang L, Pucsek AB, Cao M, Solt S, Armstrong T, Jaffee E (2015). Galectin-3 shapes anti-tumor immune responses by suppressing CD8+ T cells via LAG-3 and inhibiting expansion of plasmacytoid dendritic cells. Cancer Immunol Res.

[37] Qian W, Zhao M, Wang R, Li H (2021). Fibrinogen-like protein 1 (FGL1): the next immune checkpoint target. J Hematol Oncol.

[38] Puhr HC, Ilhan-Mutlu A (2019). New emerging targets in cancer immunotherapy: the role of LAG3. ESMO Open.

[39] Khair DO, Bax HJ, Mele S, Crescioli S, Pellizzari G, Khiabany A, Nakamura M, Harris RJ, French E, Hoffmann RM (2019). Combining immune checkpoint inhibitors: established and emerging targets and strategies to improve outcomes in melanoma. Front Immunol.

[40] Krummel MF, Allison JP (1995). CD28 and CTLA-4 have opposing effects on the response of T cells to stimulation. J Exp Med.

[41] Chen L (2004). Co-inhibitory molecules of the B7–CD28 family in the control of T-cell immunity. Nat Rev Immunol.

[42] Camacho LH (2008). Novel therapies targeting the immune system: CTLA4 blockade with tremelimumab (CP-675,206), a fully human monoclonal antibody. Expert Opin Investig Drugs.

[43] Boasberg P, Hamid O, O’Day S. Ipilimumab: unleashing the power of the immune system through CTLA-4 blockade. In: Seminars in oncology. Elsevier; 2010:440–49. 10.1053/j.seminoncol.2010.09.004 21074058

[44] Alegre M-L, Shiels H, Thompson CB, Gajewski TF (1998). Expression and function of CTLA-4 in Th1 and Th2 cells. J Immunol.

[45] McCoy KD, Le Gros G (1999). The role of CTLA-4 in the regulation of T cell immune responses. Immunol Cell Biol.

[46] Egen JG, Kuhns MS, Allison JP (2002). CTLA-4: new insights into its biological function and use in tumor immunotherapy. Nat Immunol.

[47] Motoshima T, Komohara Y, Horlad H, Takeuchi A, Maeda Y, Tanoue K, Kawano Y, Harada M, Takeya M, Eto M (2015). Sorafenib enhances the anti-tumor effects of anti-CTLA-4 antibody in a murine cancer model by inhibiting myeloid-derived suppressor cells. Oncol Rep.

[48] Leach DR, Krummel MF, Allison JP (1996). Enhancement of anti-tumor immunity by CTLA-4 blockade. Science.

[49] Yi M, Yu S, Qin S, Liu Q, Xu H, Zhao W, Chu Q, Wu K (2018). Gut microbiome modulates efficacy of immune checkpoint inhibitors. J Hematol Oncol.

[50] Cameron F, Whiteside G, Perry C (2011). Ipilimumab. Drugs.

[51] Sangro B, Gomez-Martin C, de la Mata M, Iñarrairaegui M, Garralda E, Barrera P, Riezu-Boj JI, Larrea E, Alfaro C, Sarobe P (2013). A clinical trial of CTLA-4 blockade with tremelimumab in patients with hepatocellular carcinoma and chronic hepatitis C. J Hepatol.

[52] Li Z, Ju Z, Frieri M. The T-cell immunoglobulin and mucin domain (Tim) gene family in asthma, allergy, and autoimmunity. In: Allergy and asthma proceedings. 2013:e21–6. 10.2500/aap.2013.34.3646 23406933

[53] Anderson AC, Joller N, Kuchroo VK (2016). Lag-3, Tim-3, and TIGIT: co-inhibitory receptors with specialized functions in immune regulation. Immunity.

[54] Wolf Y, Anderson AC, Kuchroo VK (2020). TIM3 comes of age as an inhibitory receptor. Nat Rev Immunol.

[55] Yoshino Y, Qi H, Kanazawa R, Sugamata M, Suzuki K, Kobayashi A, Shindo K, Matsuzawa A, Shibata S, Endo S (2019). RACK1 regulates centriole duplication by controlling localization of BRCA1 to the centrosome in mammary tissue-derived cells. Oncogene.

[56] Chen Y, Chi P (2018). Basket trial of TRK inhibitors demonstrates efficacy in TRK fusion-positive cancers. J Hematol Oncol.

[57] Wu W, Shi Y, Li S, Zhang Y, Liu Y, Wu Y, Chen Z (2012). Blockade of T im-3 signaling restores the virus-specific CD 8+ T-cell response in patients with chronic hepatitis B. Eur J Immunol.

[58] Hakemi MG, Jafarinia M, Azizi M, Rezaeepoor M, Isayev O, Bazhin AV (2020). The role of TIM-3 in Hepatocellular Carcinoma: a promising target for immunotherapy?. Front Oncol.

[59] Yan W, Liu X, Ma H, Zhang H, Song X, Gao L, Liang X, Ma C (2015). Tim-3 fosters HCC development by enhancing TGF-β-mediated alternative activation of macrophages. Gut.

[60] Tan S, Xu Y, Wang Z, Wang T, Du X, Song X, Guo X, Peng J, Zhang J, Liang Y (2020). Tim-3 hampers tumor surveillance of liver-resident and conventional NK cells by disrupting PI3K signaling. Cancer Res.

[61] Yu X, Harden K, Gonzalez LC, Francesco M, Chiang E, Irving B, Tom I, Ivelja S, Refino CJ, Clark H (2009). The surface protein TIGIT suppresses T cell activation by promoting the generation of mature immunoregulatory dendritic cells. Nat Immunol.

[62] Joller N, Kuchroo VK, Yoshimura A (2017). Tim-3, Lag-3, and TIGIT. Emerging concepts targeting immune checkpoints in cancer and autoimmunity.

[63] Joller N, Lozano E, Burkett PR, Patel B, Xiao S, Zhu C, Xia J, Tan TG, Sefik E, Yajnik V (2014). Treg cells expressing the coinhibitory molecule TIGIT selectively inhibit pro-inflammatory Th1 and Th17 cell responses. Immunity.

[64] Stanietsky N, Simic H, Arapovic J, Toporik A, Levy O, Novik A, Levine Z, Beiman M, Dassa L, Achdout H (2009). The interaction of TIGIT with PVR and PVRL2 inhibits human NK cell cytotoxicity. Proc Natl Acad Sci.

[65] Johnston RJ, Comps-Agrar L, Hackney J, Yu X, Huseni M, Yang Y, Park S, Javinal V, Chiu H, Irving B (2014). The immunoreceptor TIGIT regulates anti-tumor and antiviral CD8+ T cell effector function. Cancer Cell.

[66] Fuhrman CA, Yeh W-I, Seay HR, Lakshmi PS, Chopra G, Zhang L, Perry DJ, McClymont SA, Yadav M, Lopez M-C (2015). Divergent phenotypes of human regulatory T cells expressing the receptors TIGIT and CD226. J Immunol.

[67] Kurtulus S, Sakuishi K, Ngiow S-F, Joller N, Tan DJ, Teng MW, Smyth MJ, Kuchroo VK, Anderson AC (2015). TIGIT predominantly regulates the immune response via regulatory T cells. J Clin Investig.

[68] Minnie SA, Kuns RD, Gartlan KH, Zhang P, Wilkinson AN, Samson L, Guillerey C, Engwerda C, MacDonald KP, Smyth MJ (2018). Myeloma escape after stem cell transplantation is a consequence of T-cell exhaustion and is prevented by TIGIT blockade. Blood J Am Soc Hematol.

[69] Kong Y, Zhu L, Schell TD, Zhang J, Claxton DF, Ehmann WC, Rybka WB, George MR, Zeng H, Zheng H (2016). T-cell immunoglobulin and ITIM domain (TIGIT) associates with CD8+ T-cell exhaustion and poor clinical outcome in AML patients. Clin Cancer Res.

[70] Guillerey C, Harjunpää H, Carrié N, Kassem S, Teo T, Miles K, Krumeich S, Weulersse M, Cuisinier M, Stannard K (2018). TIGIT immune checkpoint blockade restores CD8+ T-cell immunity against multiple myeloma. Blood J Am Soc Hematol.

[71] He W, Zhang H, Han F, Chen X, Lin R, Wang W, Qiu H, Zhuang Z, Liao Q, Zhang W (2017). CD155T/TIGIT signaling regulates CD8+ T-cell metabolism and promotes tumor progression in human gastric cancer. Cancer Res.

[72] Duan X, Liu J, Cui J, Ma B, Zhou Q, Yang X, Lu Z, Du Y, Su C (2019). Expression of TIGIT/CD155 and correlations with clinical pathological features in human hepatocellular carcinoma. Mol Med Rep.

[73] Wang L, Rubinstein R, Lines JL, Wasiuk A, Ahonen C, Guo Y, Lu L-F, Gondek D, Wang Y, Fava RA (2011). VISTA, a novel mouse Ig superfamily ligand that negatively regulates T cell responses. J Exp Med.

[74] Wang J, Wu G, Manick B, Hernandez V, Renelt M, Erickson C, Guan J, Singh R, Rollins S, Solorz A (2019). VSIG-3 as a ligand of VISTA inhibits human T-cell function. Immunology.

[75] Johnston RJ, Su LJ, Pinckney J, Critton D, Boyer E, Krishnakumar A, Corbett M, Rankin AL, Dibella R, Campbell L (2019). VISTA is an acidic pH-selective ligand for PSGL-1. Nature.

[76] ElTanbouly M, Schaafsma E, Noelle R, Lines J (2020). VISTA: coming of age as a multi-lineage immune checkpoint. Clin Exp Immunol.

[77] Villarroel-Espindola F, Yu X, Datar I, Mani N, Sanmamed M, Velcheti V, Syrigos K, Toki M, Zhao H, Chen L (2018). Spatially resolved and quantitative analysis of VISTA/PD-1H as a novel immunotherapy target in human non-small cell lung cancer. Clin Cancer Res.

[78] Hong S, Yuan Q, Xia H, Zhu G, Feng Y, Wang Q, Zhang Z, He W, Lu J, Dong C (2019). Analysis of VISTA expression and function in renal cell carcinoma highlights VISTA as a potential target for immunotherapy. Protein Cell.

[79] Xie S, Huang J, Qiao Q, Zang W, Hong S, Tan H, Dong C, Yang Z, Ni L (2018). Expression of the inhibitory B7 family molecule VISTA in human colorectal carcinoma tumors. Cancer Immunol Immunother.

[80] Mulati K, Hamanishi J, Matsumura N, Chamoto K, Mise N, Abiko K, Baba T, Yamaguchi K, Horikawa N, Murakami R (2019). VISTA expressed in tumour cells regulates T cell function. Br J Cancer.

[81] Zong L, Mo S, Yu S, Zhou Y, Zhang M, Chen J, Xiang Y (2020). Expression of the immune checkpoint VISTA in breast cancer. Cancer Immunol Immunother.

[82] Loeser H, Kraemer M, Gebauer F, Bruns C, Schröder W, Zander T, Persa O-D, Alakus H, Hoelscher A, Buettner R (2019). The expression of the immune checkpoint regulator VISTA correlates with improved overall survival in pT1/2 tumor stages in esophageal adenocarcinoma. Oncoimmunology.

[83] Zhang M, Pang H-J, Zhao W, Li Y-F, Yan L-X, Dong Z-Y, He X-F (2018). VISTA expression associated with CD8 confers a favorable immune microenvironment and better overall survival in hepatocellular carcinoma. BMC Cancer.

[84] Im E, Sim DY, Lee H-J, Park JE, Park WY, Ko S, Kim B, Shim BS, Kim S-H. Immune functions as, a ligand or a receptor, cancer prognosis potential, clinical implication of VISTA in cancer immunotherapy. In: Seminars in cancer biology. Elsevier; 2021. 10.1016/j.semcancer.2021.08.008 34428551

[85] El-Khoueiry AB, Sangro B, Yau T, Crocenzi TS, Kudo M, Hsu C, Kim T-Y, Choo S-P, Trojan J, Welling TH (2017). Nivolumab in patients with advanced hepatocellular carcinoma (CheckMate 040): an open-label, non-comparative, phase 1/2 dose escalation and expansion trial. The Lancet.

[86] Okusaka T, Ikeda M (2018). Immunotherapy for hepatocellular carcinoma: current status and future perspectives. ESMO Open.

[87] Zhu AX, Finn RS, Edeline J, Cattan S, Ogasawara S, Palmer D, Verslype C, Zagonel V, Fartoux L, Vogel A (2018). Pembrolizumab in patients with advanced hepatocellular carcinoma previously treated with Sorafenib (KEYNOTE-224): a non-randomised, open-label phase 2 trial. Lancet Oncol.

[88] Wainberg ZA, Segal NH, Jaeger D, Lee K-H, Marshall J, Antonia SJ, Butler M, Sanborn RE, Nemunaitis JJ, Carlson CA (2017). Safety and clinical activity of durvalumab monotherapy in patients with hepatocellular carcinoma (HCC).

[89] Lee M, Ryoo B-Y, Hsu C-H, Numata K, Stein S, Verret W, Hack S, Spahn J, Liu B, Abdullah H (2019). Randomised efficacy and safety results for atezolizumab (Atezo)+ bevacizumab (Bev) in patients (pts) with previously untreated, unresectable hepatocellular carcinoma (HCC). Ann Oncol.

[90] Long L, Zhang X, Chen F, Pan Q, Phiphatwatchara P, Zeng Y, Chen H (2018). The promising immune checkpoint LAG-3: from tumor microenvironment to cancer immunotherapy. Genes Cancer.

[91] Gravara LD, Battiloro C, Cantile R, Letizia A, Vitiello F, Montesarchio V, Rocco D. Chemotherapy and/or immune checkpoint inhibitors in NSCLC first-line setting: what is the best approach? Future Med. 2020;9: LMT22. 10.2217/lmt-2019-0018 PMC7110571 32256708

[92] Alsuwaigh R, Lee J, Chan G, Chee CE, Choo SP (2019). Response to targeted therapy or chemotherapy following immunotherapy in patients with gastrointestinal cancers-a case series. J Immunother Cancer.

[93] Zhu X-D, Tang Z-Y, Sun H-C (2020). Targeting angiogenesis for liver cancer: past, present, and future. Genes Dis.

[94] Lee BM, Seong J (2021). Radiotherapy as an immune checkpoint blockade combination strategy for hepatocellular carcinoma. World J Gastroenterol.

[95] Kim K-J, Kim J-H, Lee SJ, Lee E-J, Shin E-C, Seong J (2017). Radiation improves anti-tumor effect of immune checkpoint inhibitor in murine hepatocellular carcinoma model. Oncotarget.

[96] Kim KJ, Lee HW, Seong J (2021). Combination therapy with anti-T-cell immunoglobulin and mucin-domain containing molecule 3 and radiation improves anti-tumor efficacy in murine hepatocellular carcinoma. J Gastroenterol Hepatol.

[97] Schoenfeld AJ, Hellmann MD (2020). Acquired resistance to immune checkpoint inhibitors. Cancer Cell.

[98] Trombetta ES, Mellman I (2005). Cell biology of antigen processing in vitro and in vivo. Annu Rev Immunol.

[99] Zaretsky JM, Garcia-Diaz A, Shin DS, Escuin-Ordinas H, Hugo W, Hu-Lieskovan S, Torrejon DY, Abril-Rodriguez G, Sandoval S, Barthly L (2016). Mutations associated with acquired resistance to PD-1 blockade in melanoma. N Engl J Med.

[100] Rosenthal R, Cadieux EL, Salgado R, Al Bakir M, Moore DA, Hiley CT, Lund T, Tanić M, Reading JL, Joshi K (2019). Neoantigen-directed immune escape in lung cancer evolution. Nature.

[101] Peng W, Chen JQ, Liu C, Malu S, Creasy C, Tetzlaff MT, Xu C, McKenzie JA, Zhang C, Liang X (2016). Loss of PTEN promotes resistance to T cell–mediated immunotherapy. Cancer Discov.

[102] Spranger S, Bao R, Gajewski TF (2015). Melanoma-intrinsic β-catenin signalling prevents anti-tumour immunity. Nature.

[103] Jenkins RW, Barbie DA, Flaherty KT (2018). Mechanisms of resistance to immune checkpoint inhibitors. Br J Cancer.

[104] ClinicalTrials.gov. A study of nivolumab in participants with hepatocellular carcinoma who are at high risk of recurrence after curative hepatic resection or ablation (CheckMate 9DX). ClinicalTrials.gov Identifier: NCT03383458. December 26, 2017.

[105] ClinicalTrials.gov. Study of pembrolizumab (MK-3475) as monotherapy in participants with advanced hepatocellular carcinoma (MK-3475-224/KEYNOTE-224). ClinicalTrials.gov Identifier: NCT02702414. March 8, 2016.

[106] ClinicalTrials.gov. Study of pembrolizumab (MK-3475) vs. best supportive care in participants with previously systemically treated advanced hepatocellular carcinoma (MK-3475-240/KEYNOTE-240). ClinicalTrials.gov Identifier: NCT02702401. February 17, 2020.

[107] ClinicalTrials.gov. Study of pembrolizumab (MK-3475) or placebo given with best supportive care in asian participants with previously treated advanced hepatocellular carcinoma (MK-3475-394/KEYNOTE-394). ClinicalTrials.gov Identifier: NCT03062358. February 23, 2017.

[108] ClinicalTrials.gov. Study of the safety, pharmacokinetics and antitumor activities of BGB-A317 in participants with advanced tumors. ClinicalTrials.gov Identifier: NCT02407990. November 17, 2021.

[109] ClinicalTrials.gov. A Phase 1/2 study to evaluate MEDI4736. ClinicalTrials.gov Identifier: NCT01693562. May 13, 2021.

[110] ClinicalTrials.gov. Phase II study of avelumab in patients with advanced hepatocellular carcinoma after prior sorafenib treatment (AvelumabHCC). ClinicalTrials.gov Identifier: NCT03389126. January 3, 2018.

[111] ClinicalTrials.gov. A study of the safety and efficacy of atezolizumab administered in combination with bevacizumab and/or other treatments in participants with solid tumors. ClinicalTrials.gov Identifier: NCT02715531. March 22, 2016.

[112] ClinicalTrials.gov. Study of cabozantinib in combination with atezolizumab to subjects with locally advanced or metastatic solid tumors. ClinicalTrials.gov Identifier: NCT03170960. May 31, 2017.

[113] ClinicalTrials.gov. IRX-2, cyclophosphamide, and nivolumab in treating patients with recurrent or metastatic and refractory liver cancer. ClinicalTrials.gov Identifier: NCT03655002. August 31, 2018.

[114] ClinicalTrials.gov. Nivolumab, fluorouracil, and interferon alpha 2B for the treatment of unresectable fibrolamellar cancer. ClinicalTrials.gov Identifier: NCT04380545. May 8, 2020.

[115] ClinicalTrials.gov. PD-1 antibody and lenvatinib plus TACE-HAIC for potential resectable HCC: a Single-arm, Phase 2 Clinical Trial (PLATIC) ClinicalTrials.gov Identifier: NCT04814043. March 24, 2021.

[116] ClinicalTrials.gov. QUILT-3.055: a study of combination immunotherapies in patients who have previously received treatment with immune checkpoint inhibitors. ClinicalTrials.gov Identifier: NCT03228667. July 25, 2017.

[117] ClinicalTrials.gov. Immune profile and prognosis of malignant liver tumors with radiofrequency ablation (RFA) therapy (RFA). ClinicalTrials.gov Identifier: NCT04707547. January 13, 2021.

[118] ClinicalTrials.gov. Combination of regorafenib and nivolumab in unresectable hepatocellular carcinoma (RENOBATE) ClinicalTrials.gov Identifier: NCT04310709. March 17, 2020.

[119] ClinicalTrials.gov. TACE and sbrt followed by double immunotherapy for downstaging hepatocellular carcinoma ClinicalTrials.gov Identifier: NCT04988945. August 4, 2021.

[120] ClinicalTrials.gov. An immuno-therapy study to evaluate the effectiveness, safety and tolerability of nivolumab or nivolumab in combination with other agents in patients with advanced liver cancer (CheckMate040). ClinicalTrials.gov Identifier: NCT01658878. August 7, 2012.

[121] ClinicalTrials.gov. An investigational immuno-therapy study of nivolumab compared to sorafenib as a first treatment in patients with advanced hepatocellular carcinoma. ClinicalTrials.gov Identifier: NCT02576509. June 26, 2020.

[122] ClinicalTrials.gov. Phase 3 study of tislelizumab versus sorafenib in participants with unresectable HCC. ClinicalTrials.gov Identifier: NCT03412773. January 26, 2018.

[123] ClinicalTrials.gov. Transarterial infusion of PD-1 antibody plus TACE-HAIC for unresectable HCC: a single-arm, phase 2 clinical Trial (AIPD-1) ClinicalTrials.gov Identifier: NCT04814030. March 24, 2021.

[124] Cui T-M, Liu Y, Wang J-B, Liu L-X (2020). Adverse effects of immune-checkpoint inhibitors in hepatocellular carcinoma. Onco Targets Ther.

[125] Cheng A-L, Hsu C, Chan SL, Choo S-P, Kudo M (2020). Challenges of combination therapy with immune checkpoint inhibitors for hepatocellular carcinoma. J Hepatol.

[126] Lai X, Friedman A (2017). Combination therapy of cancer with cancer vaccine and immune checkpoint inhibitors: a mathematical model. PLoS ONE.

[127] Abu-Sbeih H, Ali FS, Wang X, Mallepally N, Chen E, Altan M, Bresalier RS, Charabaty A, Dadu R, Jazaeri A (2019). Early introduction of selective immunosuppressive therapy associated with favorable clinical outcomes in patients with immune checkpoint inhibitor–induced colitis. J Immunother Cancer.

